# Evaluation of adhesion barrier types in a rat hepatectomy-induced adhesion model

**DOI:** 10.1186/s12893-020-00877-7

**Published:** 2020-10-27

**Authors:** Atsushi Shimizu, Miho Kai, Masako Tasaki, Naotaka Chino, Kiyoshi Hasegawa, Norihiro Kokudo

**Affiliations:** 1 Hepatobiliary-Pancreatic Surgery Division, Department of Surgery, Graduate School of Medicine, University of Tokyo, Tokyo, Japan; 2 Terumo Corporation, R&D Center, 1500 Inokuchi, Nakai-machi, Ashigarakami-gun, Kanagawa, 259-0151 Japan; 3 National Center for Global Health and Medicine, Tokyo, Japan

**Keywords:** Hepatectomy, Postoperative adhesion, Adhesion barrier material, Animal adhesion model, Hydrogel

## Abstract

**Background:**

Adhesion formation after hepatectomy creates problems for repeat hepatectomy. This study aimed to compare the effectiveness of a spray (AdSpray) and sheet adhesion barrier (Seprafilm) in a rat hepatectomy-induced adhesion model.

**Methods:**

Thirty male Sprague-Dawley rats underwent partial resection of the left lateral liver lobe. They were randomly assigned to control (*n* = 10), AdSpray (*n =* 10), and Seprafilm groups. Seven days after surgery, the animals were sacrificed, and adhesions at the hepatic resection surface were blindly evaluated.

**Results:**

In the control group, adhesions were formed in all 10 animals (100%), with a 69% adhesion extent (mean). In the AdSpray group, the incidence of adhesions (40%) and the adhesion extent (mean, 10%) were significantly lower than in the control group (incidence; *p* = 0.0147, adhesion extent; *p* = 0.0007). In the Seprafilm group, the incidence of adhesions was 70%. The adhesion extent of Seprafilm (mean, 30%) was significantly lower than in the control group (*p* = 0.0492). No significant differences were observed between the AdSpray and Seprafilm groups. As for histopathological examination, animals in the AdSpray group showed a similar healing profile to that of the control group without delayed healing and regeneration of mesothelial cells. In contrast, the Seprafilm group showed ongoing foreign body reaction to Seprafilm, and regeneration of mesothelial cells was immature at 7 days.

**Conclusions:**

Both the spray-type gel and sheet adhesion barriers significantly reduced adhesion formation after hepatectomy. The spray-type adhesion barrier caused no adverse events and induced favorable healing. These adhesion barriers may be effective in hepatectomy. Further animal studies and clinical trials are required to determine their benefits in clinical use.

## Background

Repeat hepatectomy is a highly effective treatment for primary and metastatic liver cancers and has been widely performed worldwide [1–3]. However, postoperative adhesions remain an issue in repeat hepatectomy. Postoperative adhesions that form between the remnant liver and surrounding tissues can complicate repeated resection. Such adhesions carry the risk of prolonging the operation time, increasing blood loss, or organ injuries [4, 5].

Postoperative adhesion occurs when damage to the mesothelium at a site of organ injury due to surgical insult induces fibrin deposits, thereby creating a bridge between the injured organ and surrounding tissues. Fibrin matrix is gradually replaced by fibroblasts, maturing into a fibrous band [6]. “Good” surgical procedures have been proposed as the first step for preventing postoperative adhesions. This includes strict adherence to surgical principles and minimizing surgical insult, including careful tissue handling, reliable hemostasis, avoidance of ischemia or dryness, avoidance of foreign bodies (e.g., powder in surgical gloves), and reduction of infection risk. Adhesion barriers that serve as physical barriers in injured sites have recently been used as an adjunct to surgical procedures [7, 8].

Existing adhesion barriers are available in sheet, liquid, and gel types; they have been extensively evaluated in the fields of gastroenterology and gynecology, and their benefits have been reported [9, 10]. A few studies have reported the use of adhesion barriers in hepatectomy; however, their effects in hepatectomy remain controversial [10–12]. Therefore, here, we evaluated the efficacy of two different types of adhesion barrier, namely a spray (AdSpray, Terumo Corporation, Japan) and sheet (Seprafilm, Genzyme Corporation, USA) adhesion barrier, in a rat hepatectomy-induced adhesion model.^13^

## Methods

### Animals

This experimental protocol was approved by the Animal Care and Use Committee of the Terumo Corporation. Thirty male Sprague-Dawley rats (7 weeks old, weighing between 240 and 270 g) were used. They were purchased from Charles River Laboratories Japan, Inc. (Kanagawa, Japan). They were housed in plastic cages for 10 days before surgery under standard laboratory conditions (temperature, 20–25 °C; humidity, 40–60%; and 12-h lighting cycle). They had ad libitum access to a standard laboratory diet and water.

### Study groups

Three study groups were included: control group (*n* = 10), where no treatment was applied to the hepatic resection surface and surrounding organs (hepatic resection only).; AdSpray group (*n* = 10), where AdSpray was applied at a volume of 1 mL to a 4 × 5 cm area of the hepatic resection surface and surrounding organs; and a Seprafilm group (*n* = 10), where a 4 × 5 cm piece of Seprafilm was applied to the hepatic resection surface and surrounding organs.

### Surgical procedure

The same surgeon (M.K.) performed all operations, and T.A. assisted with the operations. Rats were anesthetized with 3% isoflurane. Enrofloxacin (5 mg/kg) was administered subcutaneously for the prevention of intraoperative infections. The abdomens of the rats were shaved, disinfected, and opened by a 5-cm midline incision. The left lateral liver lobe was taken out of the abdominal cavity and resected transversely using surgical scissors to remove the end of the lobe, with an approximate width of 3.5 cm. Hemostasis of the hepatic resection surface was achieved using gauze and electrocautery. After hemostasis was achieved, the rats were randomly assigned to three groups.

For abdominal closure, the abdominal wall was sutured with 4–0 nylon sutures, and the skin was sutured using clips. The sutured area of the skin was disinfected, and the rats were returned to the housing cages. When the animals woke up, buprenorphine (0.01 mg/kg) was administered subcutaneously for pain relief. After surgery, all animals were administered an antibiotic (5 mg/kg of enrofloxacin, subcutaneously every 12 h) and an analgesic (0.01 mg/kg of buprenorphine, subcutaneously every 12 h) for 2 days.

### Evaluation of adhesion prevention

**Fig. 1:**
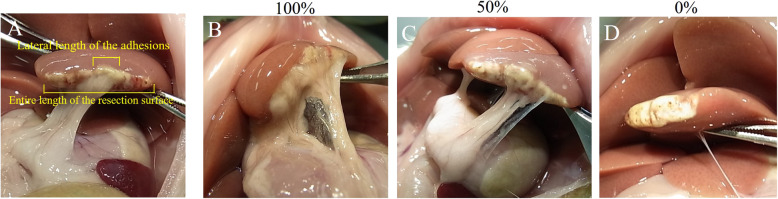
Example of adhesion extent 7 days after partial hepatectomy. **a** Measurement site of the adhesion extent. The ratio of the length of the adhesion sites to the length of the resected site was calculated (lateral length of adhesions/ entire length of resection surface). **b**, **c** Adhesions between the hepatic resection surface and greater omentum. **d** The hepatic resection surface with no adhesion

At 7 days post-surgery, the animals were euthanized by exsanguination, and necropsies were performed. After confirmation of death, a U-shaped abdominal incision was made from both costal margins, and macroscopic adhesion assessment was performed. Adhesion assessment was performed by M.T. and T.A. in a blinded manner. The ratio of the length of the adhesion sites to the length of the resected site was calculated (Fig. 1a).

### Histopathological examination

The left lateral lobe was removed without adhesiolysis and fixed in 10% neutral-buffered formalin. The left lateral lobe was divided into five parts including the resected region. The tissues were embedded in paraffin and cut into thin sections (4 μm), which were hematoxylin-eosin stained and then stained with Sirius Red for the identification of collagen fibers (fibrous tissue). Mesothelial cells were immunohistochemically stained with monoclonal mouse anti-human mesothelial cell antibodies (1:50, clone HBME-1, DAKO). After reacting with a biotin-conjugated secondary antibody, the cells were treated with horse radish peroxidase-conjugated streptavidin, followed by the addition of diaminobenzidine for color development and counterstaining with hematoxylin.

The specimens were observed microscopically by M. T and H.H. in a blinded manner. The degree of necrosis, inflammation, adherence of foam cells to the resection surface, coverage of the resection surface by a fibrous layer, regeneration of mesothelial cells, and vascular proliferation were evaluated based on grade assessment (0, nil; 1, slight; 2, mild; 3, moderate; and 4, marked). Hematoxylin-eosin stained specimens were evaluated about the necrotic area of the resection surface (necrosis) and the number of inflammatory cells (inflammation), vascular (vascular proliferation) and foam cells (adherence of foam cells to the resection surface). Sirius Red stained specimens were evaluated about the thickness of fibrous layer (coverage of the resection surface by a fibrous layer). HBME-1 stained specimens were evaluated about the coverage area and maturity of mesothelial cells (regeneration of mesothelial cells). The fibrous layer on the resection surface was graded in specimens without adhesions. In addition, the regeneration of mesothelial cells was also graded in specimens without adhesions as no mesothelial regeneration occurred in areas of adhesion.

### Statistical analyses

The incidence of adhesions was evaluated using the chi-squared test (with continuity correction). The extent of adhesion was evaluated using a Steel-Dwass multiple comparison test. Grades for necrosis, inflammation, adherence of foam cells to the resection surface, and vascular proliferation were evaluated using a Steel-Dwass multiple comparison test. Coverage of the resection surface by a fibrous layer and regeneration of mesothelial cells were compared between the AdSpray and Seprafilm groups by using the Wilcoxon paired comparison test. *P*-values < 0.05 were considered statistically significant. Statistical analyses were performed using EXSUS Ver. 7.7.1 in an SAS system (release 9.1).

## Results

### Evaluation of adhesion prevention

All animals survived until necropsy. At necropsy, no macroscopic evidence of infection or abscess was found. No residue of AdSpray or Seprafilm was observed macroscopically. Representative images of adhesion formation are shown in Fig. 1b–d.

**Fig. 2:**
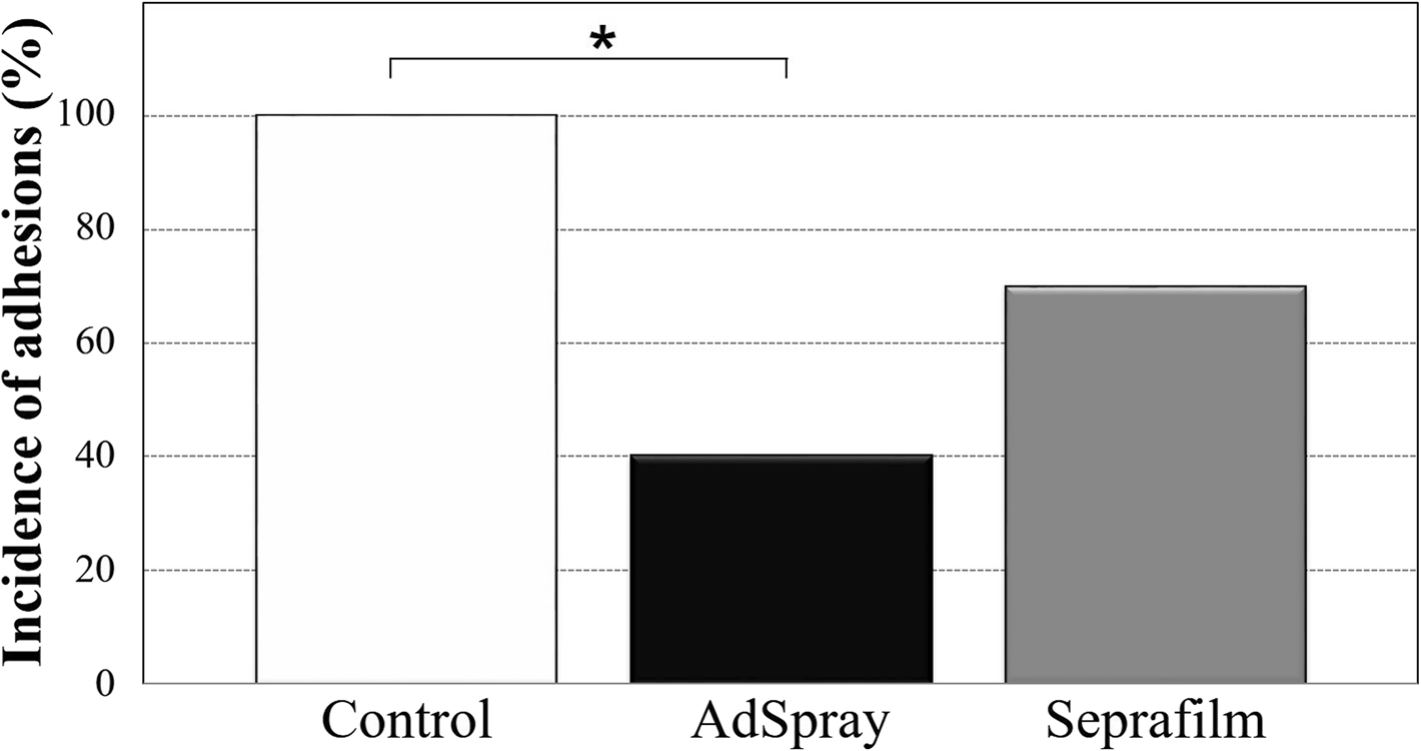
Incidence of adhesions on the hepatic resection surface. The incidence of adhesions was significantly reduced in the AdSpray group compared to the control group. The χ-squared test was performed for statistical analysis. *P < 0.05

**Fig. 3:**
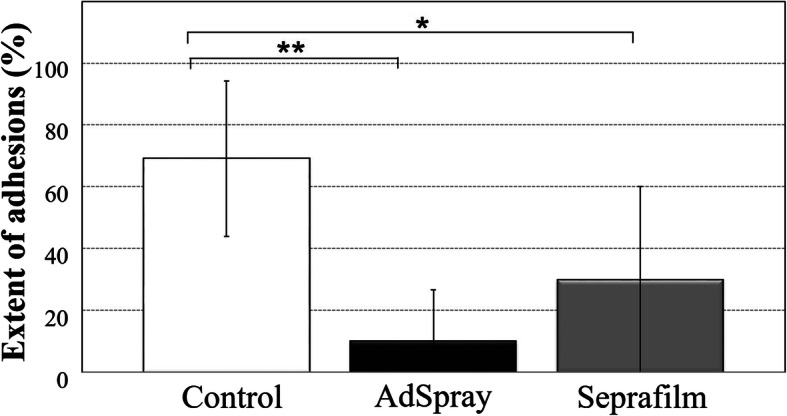
Extent of adhesions on the hepatic resection surface. The adhesion extent on the hepatic resection surface was significantly lower in the AdSpray and Seprafilm groups compared to in the control group. The Steel-Dwass multiple comparison test was performed for statistical analysis. *P < 0.05; **P < 0.001

In the control group, adhesions were formed in all 10 animals (100%), with an adhesion extent of 69 ± 25%. In the AdSpray group, adhesions were formed in four animals (40%), with an adhesion extent of 10 ± 17%. In the Seprafilm group, adhesions were formed in seven animals (70%), with an adhesion extent of 30 ± 30% (Figs. 2–3).

The incidence of adhesions was significantly lower in the AdSpray group than in the control group (*p* = 0.0147). No significant differences were found between the control and Seprafilm groups or between the AdSpray and Seprafilm groups (Fig. 2). The adhesion extent was significantly lower in the AdSpray (*p* = 0.0007) and Seprafilm (*p* = 0.0492) groups than in the control group (Fig. 3). No significant differences were observed between the AdSpray and Seprafilm groups. In all groups, adhesions to the hepatic resection surface were predominantly observed in the omentum, as were adhesions to the surrounding organs, including the middle lobe and pancreas.

### Histopathological examination

**Fig. 4:**
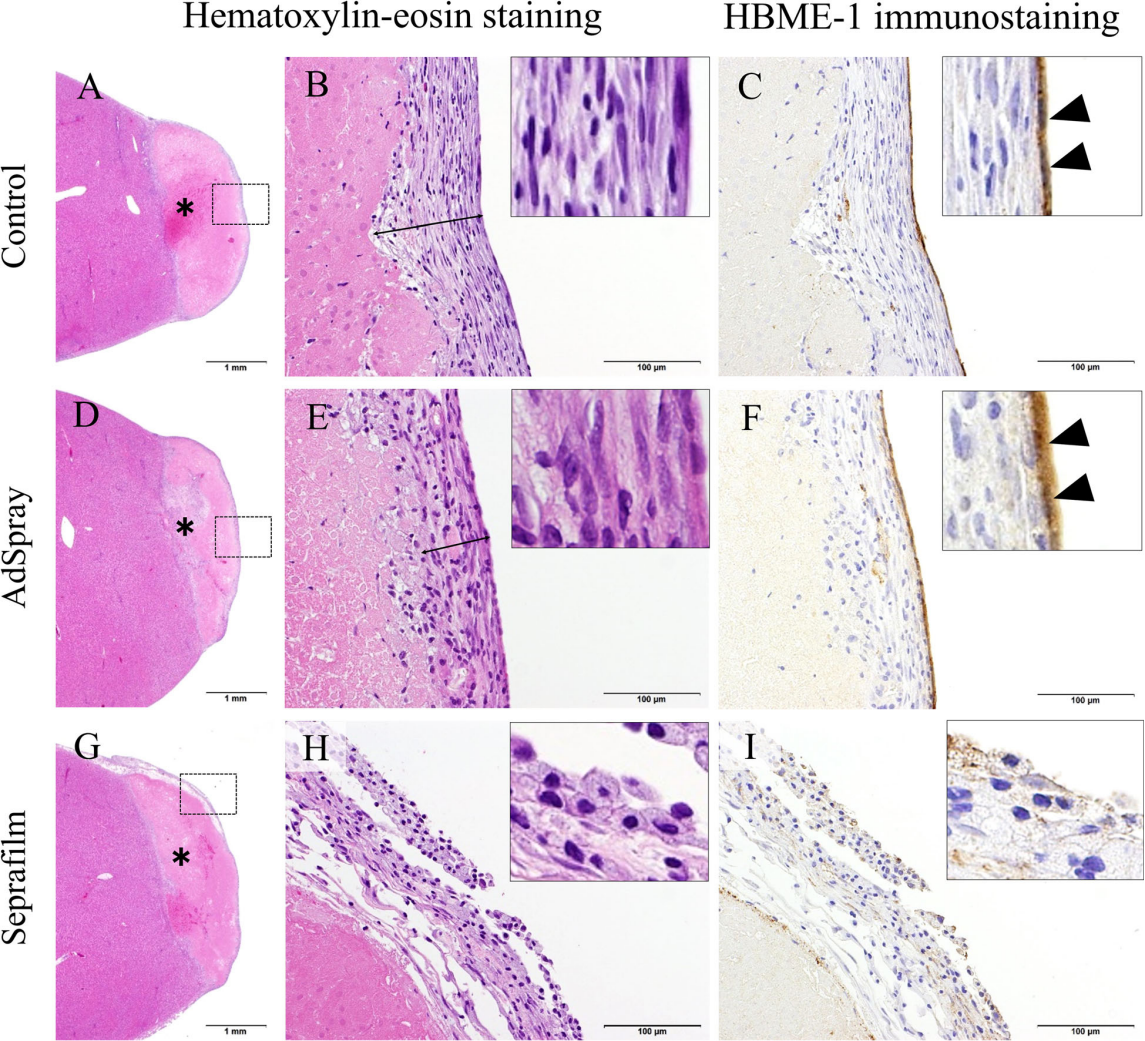
Hematoxylin-eosin staining and HBME-1 immunostaining of the hepatectomy site 7 days post-surgery in an animal without adhesions. **a** In the control group, necrosis was observed in the hepatectomy site (asterisk). **b** Higher magnifications of the box in (**a**). A fibrous layer was formed on the resection surface (arrow). **c** Continuous layer of HBME-1-positive cells (black triangles) covered the fibrous layer. **d** In the AdSpray group, necrosis was visible in the hepatectomy site (asterisk). **e** Higher magnifications of the box in (**d**). A fibrous layer formed on the resection surface (arrow). **f** Continuous layer of HBME-1-positive cells (black triangles) covered the fibrous layer. **g** In the Seprafilm group, necrosis was visible in the hepatectomy resected site (asterisks). **h** Higher magnifications of the box in (**g**). In the Seprafilm group, the marked presence of foam cells was visible on the slit-like spaces. **i** Absence of HBME-1-positive cells on the resection surface in the Seprafilm group. Bars A, D, and G = 1 mm. Bars B, C, E, F, H, and I = 100 μm

**Fig. 5:**
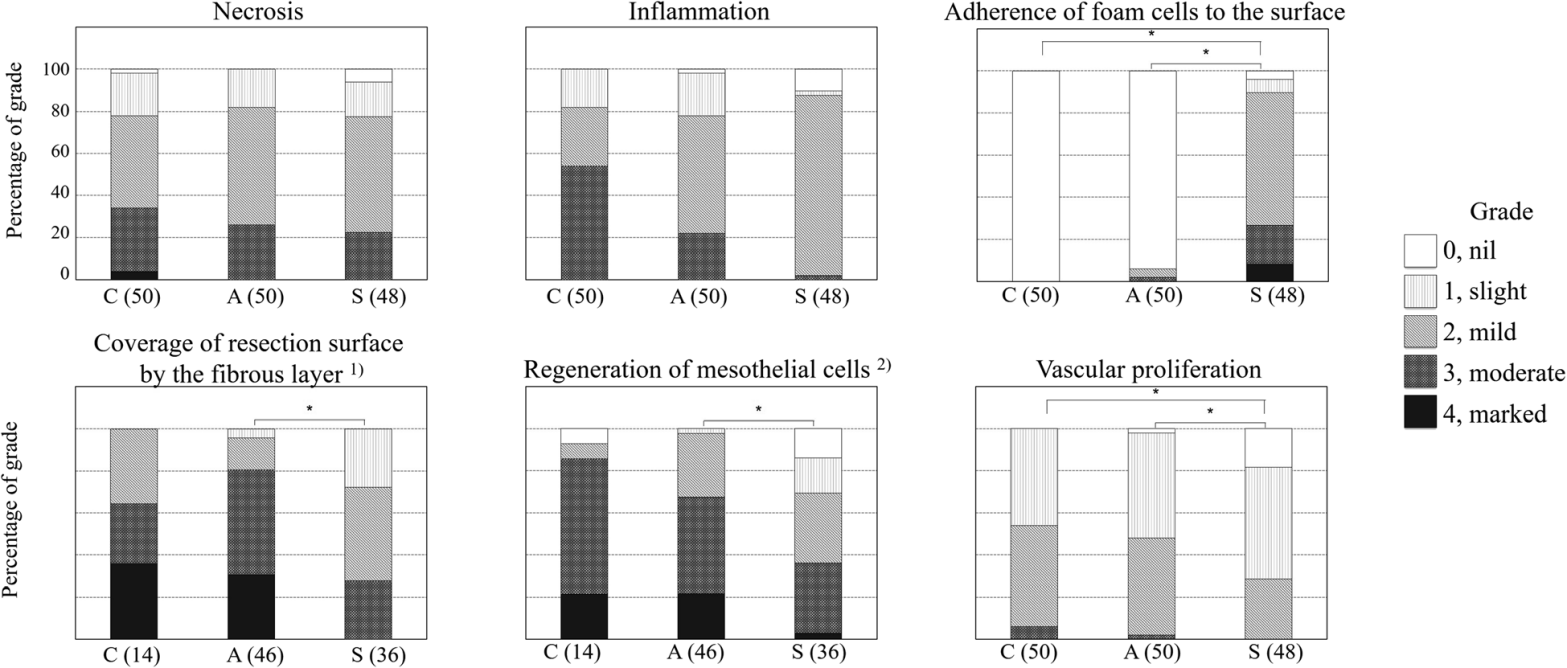
Histopathological examination of the hepatic resection surface. These graphs show the percentage of tissue reaction by grade. No significant differences were found between the control and AdSpray groups. Significant differences were found between the AdSpray and Seprafilm groups in coverage of the resection surface by the fibrous layer, vascular proliferation, adherence of foam cells to the serosa, and regeneration of mesothelial cells. Significant differences were found between the control and Seprafilm groups in vascular proliferation and foam cell adherence. A Steel-Dwass multiple comparison test was performed for statistical analysis. Values of *P* < 0.05 were considered statistically significant. 1) Coverage of the resection surface by the fibrous layer was graded in sections without adhesions. 2) Regeneration of mesothelial cells was graded in sections without adhesions as no mesothelial regeneration occurred in areas with adhesion. C, control; A, AdSpray; S, Seprafilm. The number of sections is shown in parentheses

Histopathological features of the left lateral lobe in each group are shown in Fig. 4. In the control group, a fibrous layer was formed on the resection surface and continuous layer of mesothelial cells covered the fibrous layer (Fig. 4a-c). In the AdSpray group, a fibrous layer formed on the resection surface and continuous layer of mesothelial cells covered the fibrous layer. No barrier residue was observed microscopically (Fig. 4d-f). In the Seprafilm group, slit-like spaces were found along the resection surface. Marked presence of foam cells was visible on the slit-like spaces and absence of mesothelial cells on the resection surface was observed. A barrier residue was not observed microscopically (Fig. 4g-i). Necrosis, inflammation, adherence of foam cells to the resection surface, coverage of resection surface by a fibrous layer, regeneration of mesothelial cells, and vascular proliferation were graded by each section. The coverage of the resection surface by a fibrous layer and the regeneration of mesothelial cells were graded in sections without adhesions. The percentage of tissue reaction by grade is shown in Fig. 5. The adherence of foam cells to the resection surface was not observed in the control group but was observed at a very low frequency in the AdSpray group. In contrast, the marked presence of foam cells was observed in the Seprafilm group with statistical significance (*P* < 0.05). The coverage of the resection surface by the fibrous layer and regeneration of mesothelial cells was noted in the AdSpray group with statistical significance (*P <* 0.05). Vascular proliferation was significantly lower in the Seprafilm group (*P <* 0.05). No significant differences were observed in necrosis and inflammation between the control, AdSpray, and Seprafilm groups.

## Discussion

Postoperative adhesion is an inevitable phenomenon that occurs during the healing process of surgical sites. Adhesion formation after hepatectomy is an issue, and appropriate surgical procedures and adhesion barriers for hepatectomy are needed.

Several animal models for adhesion formation after hepatectomy have been described. A previous report described a rat model induced by resection of the left lateral and median liver lobes (70% of the hepatic lobe). The model has a survival rate of 100% and an adhesion rate of 100%, representing severe adhesions [13]. In addition, mouse partial hepatectomy models were induced by the resection of a part of the left lobe using an electric scalpel. These are highly representative of the clinical setting since they expose the hepatic resection surface, leading to adhesions [14–16]. With regards to these models, we established a partial hepatectomy model in the rat. The left lateral lobe was partially resected with surgical scissors, and the bleeding was controlled with compression hemostasis and electrocautery. This model has a survival rate of 100%, while the control group achieved an adhesion rate of 100%. This model induced adhesion by partial hepatectomy in the presence of blood flow without ligation of the pedicle of the lobe; therefore, no necrosis was extended across the remnant left lateral lobe. Thus, assessing local tissue reactions and adhesions after partial hepatectomy was possible.

Adhesion barriers, such as physical barrier agents, separate injured tissues to prevent the formation of a fibrin bridge between tissues [17]. Here, we evaluated two types of adhesion barriers: Seprafilm, a sheet-type; and AdSpray, a gel-type. Seprafilm is a bioresorbable membrane that consists of hyaluronic acid and carboxymethylcellulose and forms a site-specific physical barrier [18]. Its efficacy and safety data in preclinical and clinical studies has been documented, and it has often been used in clinical practice, especially for gastrointestinal and gynecological surgeries [19, 20]. However, it has not been routinely used in liver surgery [20].

AdSpray is a bioresorbable hydrogel consisting of N-hydroxysuccinimide-modified carboxymethyl dextrin and, when applied to injured tissues using a sprayer, it forms a site-specific physical barrier [21]. A clinical trial demonstrated that it could reduce the incidence, severity, and extent of adhesions following laparotomy in gastrointestinal surgery [22]. Owing to its spray form, it is easy to use in laparoscopic surgery, and its effectiveness has been reported in a laparoscopic gynecological adhesion model in large animals [23]. To our knowledge, although there is some evidence in the literature about the adhesion prevention effect of AdSpray in gastrointestinal or gynecological surgery [21, 22, 24], evidence in hepatectomy surgery has yet to be published.

In this study, AdSpray significantly reduced adhesion incidence and extent, while Seprafilm significantly reduced adhesion extent. These results suggest that these adhesion barriers also served as barriers on the hepatic resection surface for a certain period to prevent adhesions, indicating the potential of site-specific adhesion barriers to prevent postoperative adhesion formation, even in hepatectomy surgery. The sheet-type Seprafilm and spray gel-type AdSpray have different operability characteristics. Seprafilm has the characteristics of good organ adherence and displacement resistance and is expected to create a uniform barrier on the applied area because of its sheet form [25]. However, its effect is limited to only the site it covers [20]. AdSpray provides easy access to narrow areas due to its spray formulation, and quickly creates a gel barrier on complex surfaces.

The 7-day period to necropsy was chosen for this study because, at 5–7 days post-surgery, injured surfaces in the abdominal cavity become covered by mesothelial cells [6, 17]. In this study, mesothelial regeneration was confirmed on the hepatic resection surface without adhesions at 7 days post-surgery in the control group. Animals in the AdSpray group showed a similar healing profile to the control group without delayed healing and regeneration of mesothelial cells. In contrast, in the Seprafilm group, a large number of foam cells were observed on the resection surface or serous membrane of the liver, and the mesothelial regeneration grade was lower than the AdSpray group. Seprafilm is absorbed from the application site within 7 days [18], whereas AdSpray is absorbed within 3 days [21]. Slit-like spaces observed along the resection surface in the Seprafilm group seemed to have been made by the dissolving reagents used during the histology procedure [26]. The marked presence of foam cells on the slit-like spaces indicated that the foreign body reaction to Seprafilm was still ongoing at 7 days postoperatively. The healing process was hindered by inflammatory reactions resulting from the long-term presence of foreign bodies and continuous discharge of fibrin. Accordingly, the short-term elimination of AdSpray was suggested to be advantageous to healing compared to the Seprafilm.

One limitation of our study is the animal species used. Unlike humans, the rodent liver consists of multiple lobes [27]. In addition, the rat liver is much smaller than the human liver. Larger animals are more suitable for preclinical models because they can be used to reproduce clinical surgery procedures. However, further studies and clinical trials are required to clarify the effectiveness of adhesion barriers in hepatectomy.

## Conclusions

This study evaluated both postoperative adhesion formation and its effects on healing in a model that ensured adhesion formation on the hepatic resection surface. The sheet- and spray-type adhesion barriers formed site-specific physical barriers on the hepatic resection surface and significantly reduced adhesion formation after surgery. The spray-type adhesion barriers caused no adverse events and induced favorable healing. These adhesion barriers may be effective in hepatectomy. However, further animal studies and clinical trials are required to determine their benefits in clinical use.

## Data Availability

The datasets used and/or analysed during the current study available from the corresponding author on reasonable request.
